# Comparison of Atorvastatin and Rosuvastatin in Reduction of Inflammatory Markers in Acute Coronary Syndrome

**DOI:** 10.7759/cureus.11760

**Published:** 2020-11-28

**Authors:** Salman Umrani, Waleed Jamshed, Amber Rizwan

**Affiliations:** 1 Internal Medicine, University Hospital of North Tees, Stockton-on-Tees, GBR; 2 Internal Medicine, Liaquat University of Medical and Health Sciences, Karachi, PAK; 3 Family Medicine, Jinnah Post Graduate Medical Center, Karachi, PAK

**Keywords:** acute coronary syndrome, atorvastatin, rosuvastatin, inflammatory markers

## Abstract

Introduction: Patients suffering from acute coronary syndrome (ACS) are found to have elevated levels of inflammatory markers such as high sensitivity C-reactive protein (hsCRP) and erythrocyte sedimentation rate (ESR) in their blood. These elevated inflammatory markers can lead to complications in ACS. Statins such as atorvastatin and rosuvastatin are known to reduce inflammatory markers. Our aim is to compare the efficacy of atorvastatin and rosuvastatin in reducing inflammatory markers.

Methods: This prospective, open-label, randomized trial was conducted in the cardiovascular department of tertiary care in a rural area of Pakistan. There were 128 patients diagnosed with ACS who were enrolled in the study. They were randomized into two groups, i.e. group A in which patients received 40 mg rosuvastatin daily and group B in which patients received 20 mg atorvastatin daily. hsCRP and ESR were recorded for all the patients at baseline (before starting therapy) and then again after four weeks. The results were compared between both groups.

Result: Out of 128 patients, 113 (88.2%) patients completed the study. According to this study, at the end of four weeks, rosuvastatin reduced hsCRP (p value: < 0.0001) and ESR (p value: 0.015) values significantly more when compared with atorvastatin.

Conclusion: In this study, rosuvastatin was significantly superior to atorvastatin in reducing inflammatory markers such as ESR and hsCRP in patients suffering from ACS. Cardiologists should consider using rosuvastatin rather than atorvastatin in management of patients suffering from ACS with elevated inflammatory biomarkers.

## Introduction

A fundamental role in the onset of acute coronary syndrome (ACS) involves thrombus formation on disrupted atherosclerotic lesions [1]. From initiation of atherosclerosis, through the phase of progression and ultimate complications, inflammation plays a pivotal role in the pathogenesis of atherosclerosis [2]. Previously, only markers of myocardial necrosis such as troponins were clinically used in ACS [3]. However, the cardiac biomarkers in ACS now also include markers of vascular damage evaluated by renal function tests, markers of hemodynamic instability measured by amino-terminal pro-B-type natriuretic peptide (NT-proBNP) and B-type natriuretic peptide (BNP) or markers of inflammation like high sensitivity C-reactive protein (hsCRP). These biomarkers help in earlier assessment of patients’ overall risk and also help in determining individual therapy for different ACS patients [3]. Higher baseline high-sensitivity C-reactive protein (hsCRP) levels after acute ACS are associated with adverse cardiovascular outcomes [4].

Medications used to treat patients after ACS or patients at high risk for cardiovascular events can modulate hsCRP levels. High-dose statins can accelerate the decrease in hsCRP levels after ACS [4]. In an open-label randomized trial study of the comparison of atorvastatin and rosuvastatin, it was found that both groups showed statistically significant reduction in serum hsCRP levels (p<0.0001), but rosuvastatin had a more effective role in reducing micro-inflammation in ACS patients [5]. Furthermore, in a study by Calahorra et al., recurrent atherosclerotic cardiovascular disease (ASCVD) event rates were 2.73 (95% CI: 1.63, 4.25) cases/100 person-years and 2.34 (95% CI: 1.17, 4.10) cases/100 person-years in the atorvastatin and rosuvastatin groups, respectively, which may also be related to reduction in inflammatory markers after first cardiovascular event [6].

It is important to evaluate the role of statins, particularly commonly used statins such as atorvastatin and rosuvastatin, in reducing inflammatory markers, to reduce future cardiovascular events. Despite cardiovascular events being very prevalent, currently there is limited data available from Pakistan. In this study, we compared the values of hsCRP and erythrocyte sedimentation rate (ESR) after rosuvastatin and atorvastatin therapy.

## Materials and methods

This was a prospective, open-label, randomized trial conducted in the cardiovascular department of tertiary care in a rural area of Pakistan. A total of 128 patients, diagnosed with ACS, of ages 18 years and above, and of both genders, were enrolled and randomized into two equal groups using a randomizer software. Sampling was done via consecutive convenient non-probability technique. Patients were diagnosed with ACS with the help of clinical presentation, cardiac markers, and electrocardiogram changes. All patients or their attendants gave their informed consent for inclusion before they participated in the study. The study was conducted in accordance with the Declaration of Helsinki.

After emergency treatment, group A received 20 mg rosuvastatin daily and group B received 40 mg atorvastatin once daily. Both groups also received other drugs such as anti-coagulants, beta-blockers and angiotensin-converting enzyme inhibitor at time of discharge. hsCRP and ESR were recorded for all patients at baseline (before starting statin therapy) and then again after four weeks.

Data was entered and analyzed using the IBM SPSS Statistics for Windows, Version 24 (IBM Corp., Armonk, NY). Mean and standard deviation were calculated for hsCRP and ESR levels. Dependent-T test was used to compare the values within group (baseline vs four week). Independent-T test was used to compare values between the atorvastatin and rosuvastatin group. P value of less than 0.05 meant that there is a difference between two values and the null hypothesis is not valid.

## Results

A total of 128 participants were enrolled in the study. One hundred and thirteen (113) participants (59 from the rosuvastatin group and 54 from the atorvastatin group) completed the study and 15 participants were lost to follow up. Participants who were lost to follow were not included in the final analysis. Among patients who completed the study, 61 (53.9%) participants were males and 52 (46.1%) were females.

The mean age of participants was 51 ± 12 years. There was a significant difference between baseline values and values at four weeks for hsCRP and ESR for both atorvastatin and rosuvastatin groups. At the end of four weeks, rosuvastatin reduced hsCRP (P value: < 0.0001) and ESR (P value: 0.015) values more when compared with atorvastatin (Table 1).

**Table 1:** Comparision of Atorvastatin and Rosuvastation in reducing inflammatory markers * In each group compared at four weeks from baseline (Data compared in one group between baseline and week 4) ** Inter-group comparison at four weeks (Mean change compared between both groups at week 4)

Inflammatory Marker	Groups	At Baseline	At Four Weeks	Mean Change	P-value*	P-value**
high-sensitivity C-reactive protein (hsCRP)	Rosuvastatin (n=59)	39.34 ± 10.29	18.46 ± 6.35	20.88 ± 3.94	<0.0001	<0.0001
	Atorvastatin (n=54)	38.79 ± 11.28	24.67 ± 8.45	14.12 ± 2.83	<0.0001	
erythrocyte sedimentation rate (ESR)	Rosuvastatin (n=59)	24.48 ± 13.51	18.51 ± 9.82	5.97 ± 3.69	0.007	0.015
	Atorvastatin (n=54)	23.82 ± 14.19	19.27 ± 12.01	4.55 ± 2.18	0.074	

## Discussion

Acute coronary syndrome (ACS) refers to a group of acute symptoms arising due to the decreased blood flow through the coronary arteries of the heart, leading to the death of the heart muscles. The diagnostic biomarkers not only help identify acute patients with ACS but also help determine the possible outcome of the patients.

According to our study, the drug rosuvastatin significantly reduced hsCRP and ESR in comparison to atorvastatin after four weeks. A study like ours also revealed similar results with the use of atorvastatin and rosuvastatin in ACS. Kumar et al. concluded better results with the use of rosuvastatin by significantly reducing the hsCRP and ESR [5].

Various new inflammatory markers have been identified which include cystatin C, myoglobin, cluster of differentiation 40 (CD40), pregnancy associated plasma protein-A (PAPP-A), and myeloperoxidase (MPO) [7]. The acute phase reactants may include C-reactive protein (CRP) and serum amyloid A (SAA) [8]. CRP and CD40L are the most important inflammatory biomarkers present in acute stages of ACS, thus highly valuable in the early diagnosis. The diagnosis of the death of the heart muscle involves the identification of cardiac troponin I or troponin T [7]. Troponin remains the gold standard for the diagnosis of this syndrome. The prognosis of the syndrome can be determined with the measurement of BNP and the prohormone NT-proBNP [7].

A study conducted by Leoncini predicted the protective effects of high dose rosuvastatin on patients with ACS who were at a higher risk of developing contrast-induced acute kidney injury [9]. A similar study also predicted protective effects on the kidney with the administration of high dose rosuvastatin drug. The study concluded better results with rosuvastatin in patients with high hsCRP baseline [10]. 

According to a clinical trial on atorvastatin on patients with ACS, improved results in patients receiving invasive treatments were reported thus promoting the use of high dose statins for improved results [11]. However, according to Berwanger, the routine use of atorvastatin before percutaneous coronary intervention (PCI) did not significantly reduce the rate of cardiovascular adverse effects [12]. Early treatment protocols with the use of lipid-lowering drugs revealed greater benefits in ACS [13]. A similar study has also highlighted the importance of the use of statins in reducing the mortality rates in patients with ACS [14].

The use of lipid-lowering therapy highlighted the benefits of a better outcome in ACS. β-Hydroxy β-methylglutaryl-CoA (HMG-CoA) reductase inhibitors were highly effective in lowering the inflammatory markers in ACS thus predicting better prognosis. Furthermore, the diagnostic biomarkers play a critical role in the early diagnosis as well as predicting the outcome. These inflammatory markers help identify and understand the pathophysiology behind the syndrome. The effectiveness of the biomarkers thus promotes additional research for understanding and predicting the possible disease process.

## Conclusions

In this study, it was found that rosuvastatin managed to transcend atorvastatin in lowering the levels of hsCRP and ESR markedly in ACS patients. Inflammatory markers are important markers that predicts the severity of disease and future complications, hence it is important that markers such as ESR and hsCRP are monitored regularly and therapies targed to reduce them should be used.
